# The Matron's Books

**Published:** 1907-07-13

**Authors:** 


### Nursing Administration.

### THE MATRON'S BOOKS.

### I.—PROBATIONERS' ENTRANCE ROLL AND PROGRESS BOOKS.

The importance of keeping a good record of the nursing service throughout the hospital can hardly be overestimated. It is not an easy thing to get a thoroughly good system into working order. Those who, when taking up a fresh appointment, find such a system established through the labours of their predecessors, are unusually fortunate, for there are still many laggards in modern methods of recording concise information, institutions where rough jottings are made to do duty for registers, and where the search for particulars concerning a nurse's career in past years is a process taxing the time and patience of all concerned. Posting the registers is one of the ever recurring duties of the matron, which can never be said to be done. The hospital exists in a state of flux, and at no given time can the matron fold her hands and reflect that she has wound up matters for a week in advance.

Every change which takes place in the disposition of the nurses throughout the hospital is a matter of importance to the nurse or probationer who is moved from one ward to another. Trifling as the variations may appear in her work, taken all together they make up her training, and it is a matter of common justice to each individual that a clear record of all she has done shall be available for reference. The registers which it will be found indispensable to keep are three in number. 1. Entrance Roll for Probationers. 2. Probationers' Register. 3. Sisters' and Nurses' Register.

1. The entrance roll for probationers is a record in concise form of useful particulars regarding the past of all the probationers who enter the service of the hospital, whether they stay three days or ten years. A probationer may come on trial, prove unsatisfactory in every particular and be dismissed, yet from the mere fact of her admission, the hospital is liable to be called upon for an account of her at some future time, when it may be of great importance to be able to produce an entry showing how long the person in question was in the service of the institution, and for what reason she was discharged. The following facts should be obtained:—

Name.
Date of entering.
Date of leaving, or of engagement. (If engaged as regular probationer the folio in book 2 is appended.)
Cause of leaving.
Age. Date. Place of birth.
Single or widow.
Religious denomination.
Educated at.
Previous occupation.
Recommended by 1.
Recommended by 2.
Medical certificate signed by.
Parents living. Father's occupation.
Home address.

Book 2 is the most important of all. The Register of Probationers is likely to be constantly in request, and the manner in which it is kept has more to do with the general standard of training in the hospital than is often understood. It should be so planned as to give at a glance the whole of the probationer's career in the hospital. If too crowded with detail it misses its aim. If too scanty it may be absolutely misleading. The form in use at the London Hospital and put in evidence before the Select Committee on the Registration of Nurses is admirable. The first part of the page is occupied with a record of the pupil probationer's career at Tredegar House, the preliminary training school belonging to the London hospital. The second part contains the following particulars:—

| Wards where Placed. | Dates. From | To | Day or Night Duty. | Special Cases. Remarks. |
|---|---|---|---|---|
| Crossman ... ... | March 24 | March 28 | Night | Ovariotomy |

The names of the wards of course carry with them the nature of the work, such as surgical, medical, children, etc.

Another good form is that in use at King's College Hospital.

*Record of Work During the Three Years' Period of Training.*

| Surgical Work. | Days. | Nights. | Medical Work. | Days. | Nights. | Special Cases. | Illness. Nature of |
|---|---|---|---|---|---|---|---|
| Male surgical ...<br>Female surgical<br>Children's surgical<br>Ophthalmic ...<br>Ear cases ......... | | | Male medical...<br>Female medical<br>Children's medical<br>Gynæcological<br>Throat ............<br>Midwifery ...... | | | | |
| Total ... | — | — | Total ... | — | — | | |

Total of surgical work.
Total of medical work.
Illness.
Off-duty.
Holidays and special leave.

This latter form, it will be seen, is compiled at the conclusion of the three years' training from particulars collected from such a daily register as is given above.

At St. George's Hospital the Register of Probationers gives space for a report from each sister in whose ward part of the training has been received, together with the special nursing duties learned in the ward. This is excellent, as it is far easier to appraise the value of a nurse's work and disposition when the report of a succession of superior officers are studied side by side.

